# A framework to predict the applicability of Oncotype DX, MammaPrint, and E2F4 gene signatures for improving breast cancer prognostic prediction

**DOI:** 10.1038/s41598-022-06230-7

**Published:** 2022-02-09

**Authors:** Kevin Yao, Chun-Yip Tong, Chao Cheng

**Affiliations:** 1 Department of Electrical and Computer Engineering, Texas A&M University, College Station, TX USA; 2 Department of Medicine, Baylor College of Medicine, Houston, TX 77030 USA; 3 Dan L Duncan Comprehensive Cancer Center, Baylor College of Medicine, Houston, TX 77030 USA; 4 Institute for Clinical and Transcriptional Research, Baylor College of Medicine, Houston, TX 77030 USA

**Keywords:** Cancer, Genome informatics

## Abstract

To improve cancer precision medicine, prognostic and predictive biomarkers are critically needed to aid physicians in deciding treatment strategies in a personalized fashion. Due to the heterogeneous nature of cancer, most biomarkers are expected to be valid only in a subset of patients. Furthermore, there is no current approach to determine the applicability of biomarkers. In this study, we propose a framework to improve the clinical application of biomarkers. As part of this framework, we develop a clinical outcome prediction model (CPM) and a predictability prediction model (PPM) for each biomarker and use these models to calculate a prognostic score (*P*-score) and a confidence score (C-score) for each patient. Each biomarker’s *P*-score indicates its association with patient clinical outcomes, while each C-score reflects the biomarker applicability of the biomarker’s CPM to a patient and therefore the confidence of the clinical prediction. We assessed the effectiveness of this framework by applying it to three biomarkers, Oncotype DX, MammaPrint, and an E2F4 signature, which have been used for predicting patient response, pathologic complete response versus residual disease to neoadjuvant chemotherapy (a classification problem), and recurrence-free survival (a Cox regression problem) in breast cancer, respectively. In both applications, our analyses indicated patients with higher C scores were more likely to be correctly predicted by the biomarkers, indicating the effectiveness of our framework. This framework provides a useful approach to develop and apply biomarkers in the context of cancer precision medicine.

## Introduction

In the era of precision medicine, biomarkers will be heavily implemented to improve diagnosis, prognosis, and treatment of human diseases^1–4^. Cancer is a heterogeneous disease with tumors from patients of the same cancer type associated with different sets of somatic mutations, gene expression changes, epigenetic changes, and other genomic aberrations^5–7^. This inter-tumoral heterogeneity introduces dramatic variations among patients regarding clinical phenotypes, prognosis, and sensitivity to therapeutic treatment^8,9^. In order to stratify patients to improve treatment efficacy and reduce adverse effects, a large number of biomarkers have been proposed from previous studies to predict prognosis and patient response to treatment^10–14^.

The Oncotype DX assay is one genomic test that has been widely used clinically to predict the recurrence risk of patients with estrogen-receptor-positive (ER+) breast cancer^15^. In this genomic assay, the expression levels of 16 marker genes and 5 control genes are measured to calculate a recurrence score, that can be used to stratify patients into three prognostic groups with high-, intermediate- and low-risk. It has been shown that high-risk patients are more likely to benefit from and should be treated by adjuvant chemotherapy, whereas low-risk patients do not benefit from chemotherapy and should thus not be treated to avoid side effects^16^. It has also been shown that breast cancer patients with higher Oncotype DX scores are more likely to achieve pathologic complete response (pCR) when treated by neoadjuvant therapy before surgery^16^. The American Society of Clinical Oncology (ASCO) and the National Comprehensive Cancer Network (NCCN) have recommended the application of the Oncotype DX assay to aid breast cancer clinical decisions^12,15^. Another example of a successful genomic test is MammaPrint, which has been used to predict the recurrence risk of patients with breast cancer irrespective of estrogen receptor status based on the expression of a panel of 70 genes^17,18^.

Despite the success of these two genomic tests, the heterogeneity between tumors from different patients limits the applicability of many biomarkers and genomic assays, making them effective only in a subgroup of patients. Indeed, the Oncotype DX assay can only be applied to ER+ breast cancer^19^. While MammaPrint can be applied to both ER+ and ER- breast cancer, it has been suggested that to be eligible for this assay a breast tumor sample should be from Stage I or II with tumor size less than 5.0 cm^17^. Meanwhile, more than 99% of published cancer biomarkers or genomic assays have failed to enter clinical practice^20^. At least some of these biomarkers failed because their applicability is unclear—they are effective for patients with certain over-represented features in the discovery data but cannot be reproduced in other data consisting of a different population of patients. In the field of biomarker development, there is thus a lack of effective methods based on clinical and genetic information to determine the applicability of biomarkers and predict for which patients a biomarker is most likely to be accurate.

In this study, we develop a new framework to apply biomarkers that will jointly calculate a pair of scores for each patient: one indicates the clinical outcome predicted by the biomarker (prognostic score, *P*-score), while the other indicates the applicability of this biomarker to this patient (confidence score, C-score). This framework is based on the rationale that most biomarkers do not apply to all patients, and the applicability of them varies between patients depending on their clinical (e.g., age, stage) and genomic (e.g., somatic mutations) features. We note that our methods are distinct from confidence prediction scores that models inherently have because our confidence score considers the effect of clinical features. We use three multi-gene signatures, including Oncotype DX, MammaPrint, and an E2F4 signature^21–23^, to demonstrate the potential of this framework to change the application of biomarkers. We use these signatures to construct classification models to predict patient response to neoadjuvant (pCR vs. RD, residual disease), and Cox regression models to predict patient recurrence-free survival in breast cancer. Our results indicate that patients with higher C-scores—patients to whom a biomarker is more applicable—are associated with higher prediction accuracy. We recommend that this framework should be used during the application of all prognostic and predictive biomarkers. Other than cancer, it may also be extended to other human diseases.

## Results

### A framework to jointly predict clinical outcome and model applicability

Due to the heterogeneous nature of cancer, most biomarkers are only applicable to a subset of tumors with a particular set of clinical and genomic features. A large proportion of cancer biomarkers are proposed to predict the clinical outcomes of patients under standard or a particular therapeutic treatment. Here we call the models that are developed to apply biomarkers clinical outcome prediction models (CPMs). Depending on the type of clinical outcome variable, a CPM can be formularized as a classification problem (Classification-CPM) or a survival analysis (Cox regression-CPM). The former is used to classify patients into different groups, such as recurrence versus non-recurrence within five years, and pathological complete response (pCR) versus residual disease (RD) after neoadjuvant therapy. The latter is used to predict the survival times (e.g., recurrence-free survival times) of patients.

**Figure 1:**
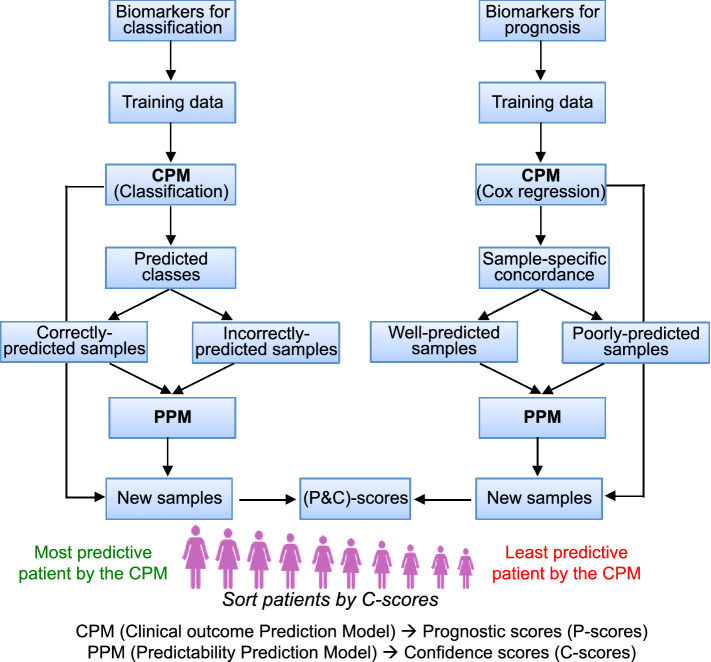
A schematic diagram of our framework for applying cancer biomarkers. Two models will be applied jointly to predict the clinical outcome of cancer patients and provide a confidence score of the prediction. The clinical outcomes of patients with higher confidence scores are more likely to be correctly predicted. This framework applies to classification (left side) and survival prediction (right side) problems.

As shown in Fig. 1, we propose a framework to determine the applicability of CPMs to different tumor samples. First, we apply a CPM to a benchmark dataset, and by comparing the predicted results with known clinical outcomes, divide tumor samples into correctly versus incorrectly predicted groups for Classification-CPMs, or well- versus poorly-predicted groups for Cox regression-CPMs. We then identify the genomic or clinical features that are associated with the applicability of CPMs, namely, features that discriminate between the two groups. Finally, we construct a classification model (denoted as a predictability prediction model, PPM) based on these features to determine the predictability of tumor samples by a CPM.

Following this procedure, a pair of models, a CPM and a PPM, is developed to implement a biomarker. For each tumor sample, the CPM calculates a prognostic score (*P*-score) to predict the clinical outcome of the patient, and the PPM calculates a confidence score (C-score) to indicate the reliability of this prediction. Based on the C-scores, patients can be sorted with higher clinical outcome prediction accuracy expected for those with higher C-scores.

In the next two sections, we will demonstrate the efficacy of this framework using three multi-gene signatures developed for predicting clinical outcomes in breast cancer. In one section, we will apply these signatures to predict patient response to neoadjuvant therapy using a combination of PPMs and classification-CPMs. In the other section, we will apply them to predict recurrence-free survival of patients using a combination of PPMs and Cox regression-CPMs.

### Application to classification models

To test the applicability of our framework to classification problems, we used the Hatzis breast cancer microarray dataset, which contains gene expression profiles from pre-treatment tumor biopsies of 508 patients treated with neoadjuvant taxane-anthracycline chemotherapy^24^. After treatment, these patients were classified as pCR or RD based on their clinical response. The dataset consists of a discovery dataset and a validation dataset with 310 and 198 patients, respectively.

**Figure 2:**
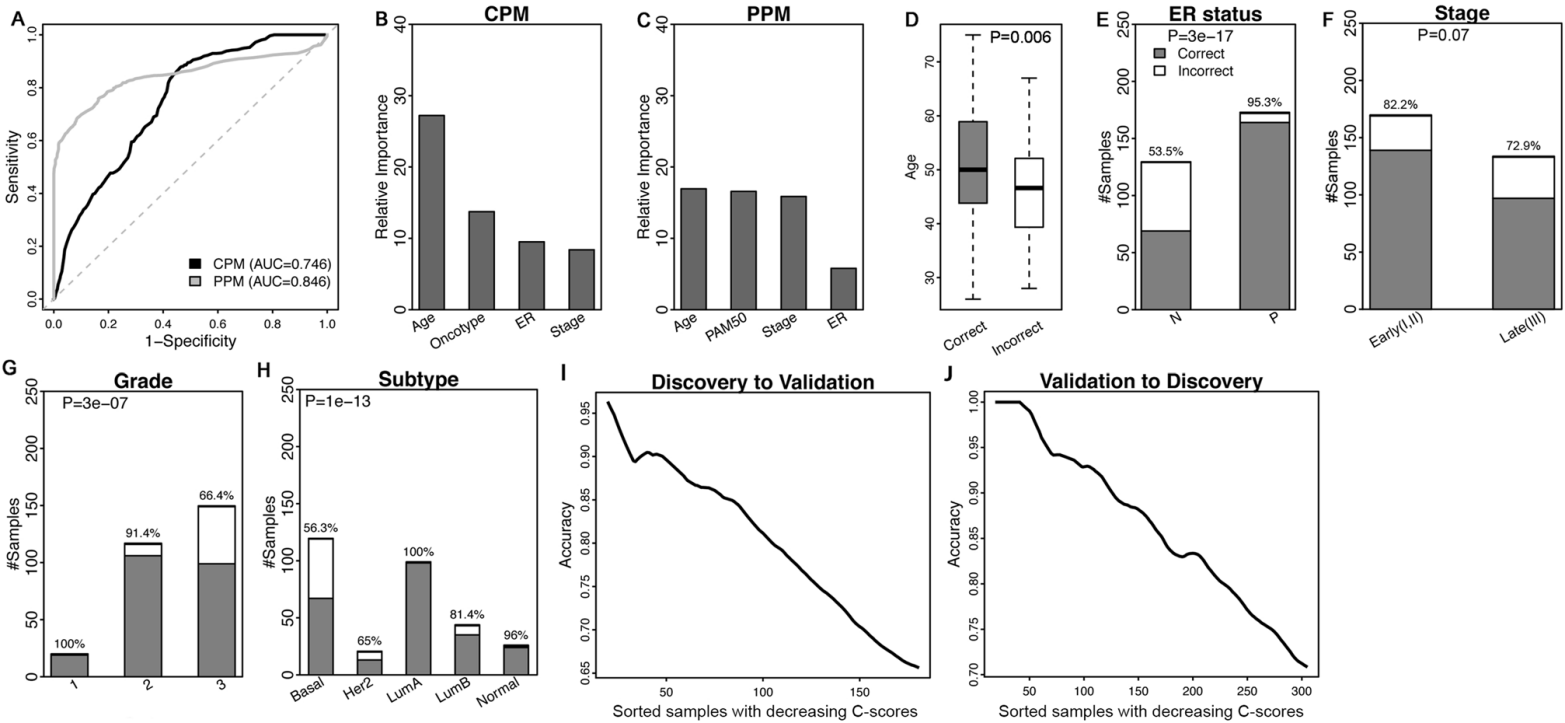
Predicting breast cancer patient response to neoadjuvant chemotherapy by using the Oncotype DX biomarker. To apply this biomarker, a clinical outcome prediction model (CPM) is used to predict the probability of pathologic complete response (pCR), and a predictability prediction model (PPM) is used to predict the applicability of this CPM to each patient. Both models are developed using random forest method. (**A**) The ROC curves of the CPM and the PPM calculated using the Hatzis discovery data. (**B**) The relative importance of predictors included in the CPM model. (**C**) The relative importance of predictors included in the PPM model. (**D**) Old patients are more likely to be corrected predicted. (**E**) ER+ patients are associated with higher prediction accuracy than ER- patients. (**F**) Low stage patients are more likely to be correctly predicted. (**G**) Low grade patients are more likely to be correctly predicted. (**H**) Association of molecular subtypes with prediction accuracy. (**I**) CPM and PPM are trained from the discovery data and then applied to predict the response and confidence score of patients in the validation cohort. Patients with higher confidence scores are more likely to be correctly predicted. Patients are sorted in the decreasing order of their confidence scores from the PPM, and the average prediction accuracy of the CPM in the top N patients were calculated from *N* = 1 to all patients. The curve was smoothed by averaging values within a sliding window of size 20. (**J**) Apply CPM/PPM trained from the validation data to predict patients in the discovery cohort.

Using the discovery cohort, we constructed a random forest model (CPM) to classify pCR and RD patient groups based on the Oncotype DX scores of each patient, as well as several clinical variables including age, ER status and tumor stage. We noted that the AUC scores of the models generally increased or was comparable when clinical variables are introduced (Oncotype DX AUC: 0.746, MammaPrint AUC: 0.748, E2F4 AUC: 0.742), compared to when the Oncotype DX score (AUC: 0.731), MammaPrint score (AUC: 0.743), or E2F4 score (AUC: 0.708) are used as individual predictors (Suppl. Fig. 1). The clinical variables used as predictors are readily available in datasets and clinics, so we performed further analyses with clinical variables included in the model. This model predicted patient response with a fairly good accuracy (AUC = 0.746) as estimated by cross-validation (Fig. 2A). Out of the 305 patients (5 patients were excluded for the lack of ER information), 236 (77.4%) were correctly predicted by the CPM. We then constructed another random forest model (PPM) to classify patients that were correctly and incorrectly predicted. In this PPM, we used the following clinical variables as predictors: age, ER status, tumor stage, and PAM50 molecular subtype. Interestingly, cross-validation results indicated a higher accuracy for the PPM (AUC = 0.846) than the CPM (Fig. 2A), indicating that the applicability of the CPM is strongly determined by patient clinical features and therefore is highly predictable by the PPM. In Fig. 2B and C, we compared the relative importance of each predictor in the CPM and the PPM. As shown, to predict patient response to neoadjuvant therapy, the age is the most important variable followed by Oncotype score (Fig. 2B). In contrast, age, PAM50 subtype and stage contribute equally to the prediction of CPM applicability to patients in the PPM (Fig. 2C).

The Oncotype DX assay is recommended for ER+ but not ER- breast cancer. However, other clinical variables that determine its applicability have not been carefully investigated.

Here we systematically investigated the association of CPM prediction accuracy with all clinical and genomic variables available from the Hatzis data. As shown in Fig. 2D–G many clinical features were correlated with predictability of patients by the CPM, in which Oncotype DX score was included as one of predictors. Notably, 95.3% of ER+ patients were correctly predicted while this was the case in only 53.5% of ER- patients, consistent with the reported applicability of Oncotype DX assay^19^ (Fig. 2E). It was also notable that the CPM was less applicable to tumor samples with higher grade or stage (Fig. 2F and G). The applicability of the CPM also depended on the molecular subtypes with better performance in the Lum A and Normal-like tumors, whereas the prediction accuracy for Basal-like and Her2-enriched subtypes was significantly lower (Fig. 2H). These results indicated that recommending the application of a biomarker simply based on a single or subset of clinical variables may not be valid. To better implement biomarkers, a more sophisticated model like the PPM proposed here may be required to quantitatively measure the applicability of biomarkers to different tumor samples.

We then applied the CPM and the PPM trained using the discovery dataset to the validation cohort of the Hatzis data. For each patient in the validation cohort, we calculated a *P*-score and a C-score that indicated the likelihood of this patient to achieve pCR and the confidence of the prediction, respectively. A patient was predicted to be pCR if they had a *P*-score > 0.5, and RD, otherwise. We sorted patients based on their C-scores and calculated the average prediction accuracy in the top patients. Our results indicate that patients with higher C-scores were associated with higher accuracy, with average accuracy decreasing from 95 to 65% (Fig. 2I). The same results were obtained when we trained the CPM/PPM using the validation cohort and test in the discovery cohort (Fig. 2J).

**Figure 3:**
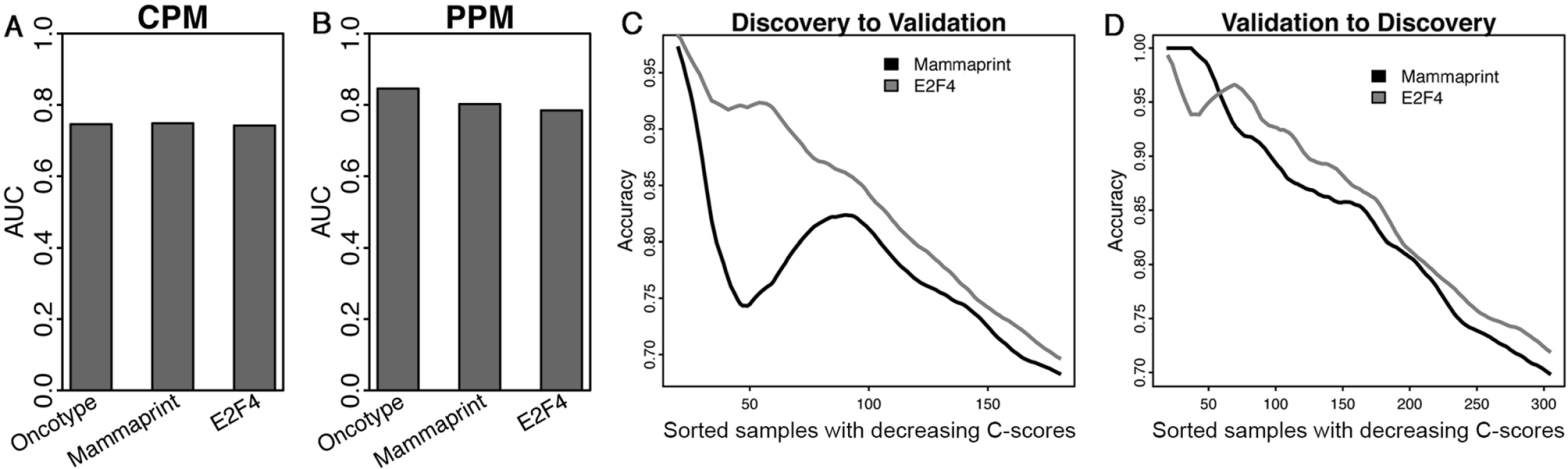
Applying MammaPrint and an E2F4 signature to predict patient response to neoadjuvant chemotherapy in breast cancer. (**A**) The prediction accuracy of the CPM models is based on the Oncotype DX, the MammaPrint, and the E2F4 scores, respectively, and (**B**) the prediction accuracy of the corresponding PPMs. AUC scores were estimated based on tenfold cross-validation using the Hatzis discovery data. (**C**) Application of the CPM/PPMs trained from the discovery data to patients in the validation cohort, and (**D**) vice versa. Patients are sorted in the decreasing order of their confidence scores from the PPM, and the average prediction accuracy of the CPM in the top N patients was calculated from *N* = 1 to all patients. The curve was smoothed by averaging values within a sliding window of size 20.

We next examined two other gene signatures for clinical outcome prediction in breast cancer, the MammaPrint and the E2F4 signatures. Both signatures have been shown to be predictive of recurrence risk of patients and patient response to neoadjuvant therapy in breast cancer. We applied the random forest method to construct the CPMs for MammaPrint and E2F4 separately by using the MammaPrint/E2F4 score and the same set of clinical variables (age, ER status and stage) as included in the CPM for the Oncotype DX signature. Similarly, we constructed the PPMs for them using age, ER status, stage, and PAM50 subtype as predictors. The CPMs and PPMs for Oncotype DX, MammaPrint, and E2F4 signatures all achieved comparable AUC scores as shown in Fig. 3A and B. The MammaPrint and E2F4 CPMs also predict more accurately when patients are older, are ER positive, have low grade, and have the Luminal A or Normal subtype (Suppl. Figs. 2, 3), which is very similar to the Oncotype DX CPM. The CPMs and PPMs were then trained using the discovery cohort and then used to calculate *P*-scores and C-scores for patients in the validation cohort, and vice versa. As shown in Fig. 3C and D, we obtained a similar trend as observed from the Oncotype DX analysis–patients with higher C-scores are more likely to be accurately predicted.

**Figure 4:**
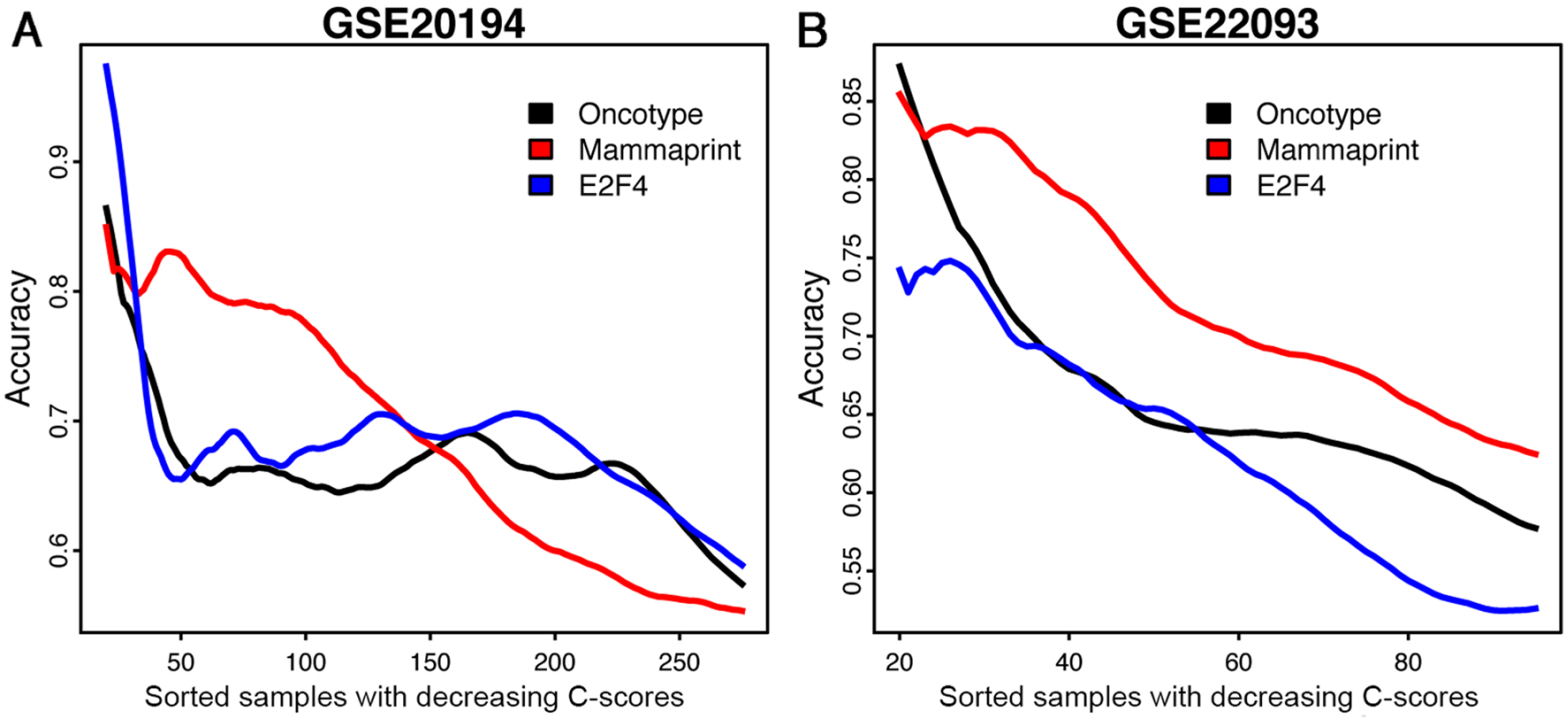
Applying the CPM/PPMs trained from the Hatzis data to two independent neoadjuvant breast cancer datasets, (**A**) GSE20194 and (**B**) GSE22093. For each patient a prognostic score (*P*-score) and a confidence score (C-score) were predicted by the CPM and the PPM, respectively. Patients were sorted in the decreasing order of their C-scores, and the average prediction accuracy of the CPM in the top N patients was calculated from *N* = 1 to all patients. The curve was smoothed by averaging values within a sliding window of size 20.

Following that, we applied the CPMs and PPMs trained from the Hatzis data (including patients from both discovery and validation cohorts) to two independent neoadjuvant therapy microarray datasets (GSE20194 and GSE22093). In both datasets, we confirmed that our framework could be used to determine the applicability of clinical outcome prediction models to each patient based on their clinical features (Fig. 4).

### Application to survival prediction models

**Figure 5:**
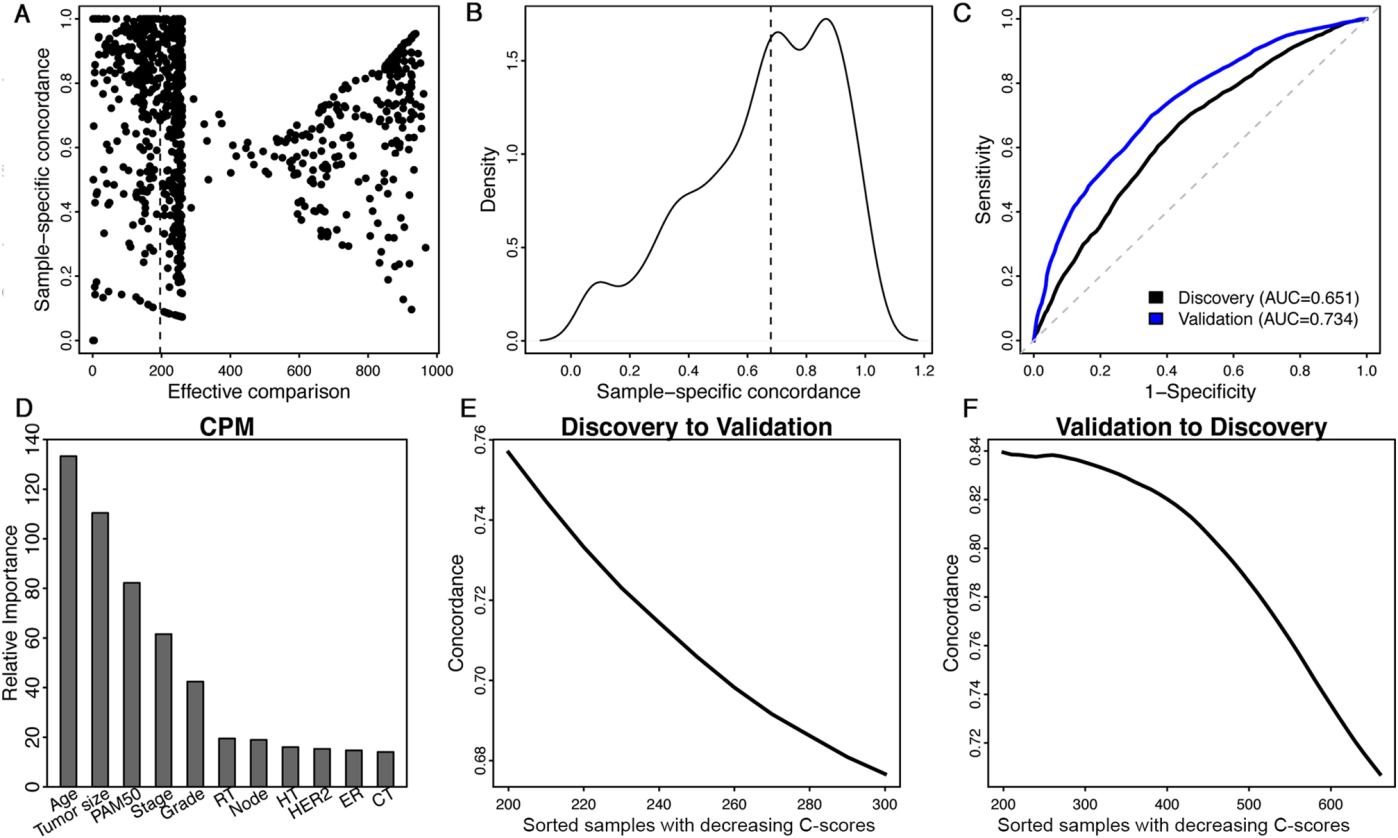
Predicting the recurrence-free survival of patients with breast cancer using the Oncotype DX biomarker. (**A**) Patients vary substantially in their number of effective comparisons and sample-specific concordance scores resulting from the Cox regression model (CPM) using the Oncotype DX score as the single predictor. (**B**) The distribution of sample-specific concordance scores of patients. Results shown in A and B are based on the Curtis discovery data. (**C**) The prediction accuracy of the PPM was estimated from tenfold cross-validation using the Curtis discovery and validation data. (**D**) The relative importance of predictors used in the PPM (discovery data). (**E**) Application of the CPM/PPMs trained from the discovery data to patients in the validation cohort, and (**F**) vice versa. Patients are sorted in the decreasing order of their confidence scores, and the average concordance in the top N patients was calculated from *N* = 1 to all patients. The curve was smoothed by averaging values within a sliding window of size 200.

Oncotype DX has been widely used to predict the recurrence risk of patients with breast cancer. In this section, we used the Curtis breast cancer dataset to demonstrate how to apply our framework to improve prognostic prediction. The Curtis dataset includes a discovery cohort and a validation cohort, containing tumor gene expression profiles from 997 and 995 patients with breast cancer as well as detailed clinical information^25^. We calculated the Oncotype DX score for each sample, and constructed a univariate Cox regression model (CPM) to predict the recurrence-free survival of patients in the discovery cohort. The concordance index of this model is 0.679. That is, for 67.9% of all effective patient pairs (patient pairs for which survival times can be compared with certainty, which is if both patients have experienced the event, or one patient has experienced the event before the other patient is censored), the patient with the longer survival time is also predicted by the Cox regression model to have longer survival time. The prediction accuracy associated with different patients varies substantially. We calculated a sample-specific concordance for each patient as the fraction of correct predictions in all effective pairwise comparisons involving this patient. Figure 5A shows the number of effective comparisons and sample-specific concordance associated with each patient in the Curtis discovery cohort. As shown, some patients associated with a small number of effective comparisons due to short follow-up time and absence of recurrence event during follow-up. Even for patients with a large number of effective comparisons, the sample-specific concordances showed a high variation, ranging from 10 to 95%. We chose patients with at least 20% of comparisons being effective (997*0.2 = 199), and divided them into a well-predicted group and a poorly-predicted group, using the overall concordance 0.679 as the cut-off value (Fig. 5B).

Following that, we constructed a random forest model to classify well-predicted versus poorly-predicted patients (PPM). In the model, we include the following clinical variables: age, tumor size, grade, stage, lymph node status, PAM50 subtypes, ER-status, HER2 status, chemotherapy, radiotherapy, and hormone therapy. The prediction accuracy of this PPM was estimated using cross-validation, and an AUC score of 0.651 and 0.734 were achieved in the discovery and the validation cohort, respectively (Fig. 5C). Clinical variables with high relative importance in the model included age, tumor size, PAM50 subtype, stage and grade, whereas ER, HER2, lymph node, and treatment are associated with low relative importance (Fig. 5D).

We trained the CPM (the univariate Cox regression using Oncotype DX score as the predictor) and the PPM (the above-described random forest model) using the discovery cohort from the Curtis data and applied them to the validation cohort. Although the two cohorts both have similar number of patients (997 in discovery and 995 in validation), the discovery dataset is relatively complete while many clinical variables have missing information in the validation dataset. We thus excluded patients without survival time, event status, age, tumor size, grade, stage, lymph node status, PAM50 subtypes, ER-status, HER2 status, chemotherapy, radiotherapy, or hormone therapy information. For the remaining patients in the validation cohort, we calculated a *P*-score, a C-score, and sample-specific concordance. We then sorted all patients in decreasing order of their C-scores, and calculated the average concordance in the top ranked patients. As shown in Fig. 5E, we observed a clear trend that patients with higher C-scores were more correctly predicted from their Oncotype DX scores by the CPM. Similarly, we trained the CPM/PPM using the validation cohort, applied them to patients in the discovery cohort, and obtained consistent results (Fig. 5F). These results indicate that the applicability of Oncotype DX varies substantially between patients, which can be correctly determined using the PPM based on the patient clinical features.

To account for potential nonlinear associations between predictors and outcome, we repeated the above analyses using a random survival forest model, which is a modified random forest model that accounts for censored survival data. The random survival forest model achieved a higher concordance index of 0.763 in the Curtis discovery dataset. We likewise also constructed a random forest model to classify well-predicted versus poorly-predicted patients (PPM) using 0.763 as the cutoff between good and bad prediction. This model was trained on the Curtis discovery cohort and achieved an AUC of 0.674. When applied to the Curtis validation cohort, it achieved an AUC of 0.649. Patients sorted with decreasing C-scores also clearly showed decreasing concordances when predicted by the CPM (Suppl. Fig. 4). Similar trends were observed when the CPM and PPM were initially trained in the Curtis validation cohort and applied to the discovery cohort. This result is consistent with that of the Cox regression model.

**Figure 6:**
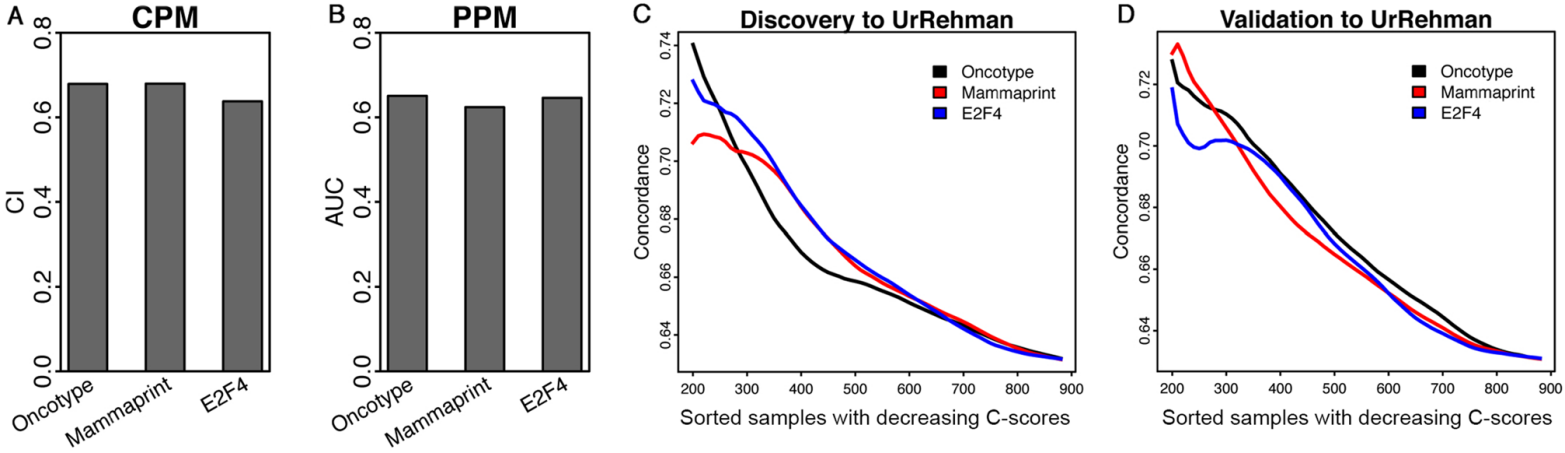
Applying the CPM/PPMs trained from the Curtis data to the Ur-Rehman data. (**A**) The overall concordance indexes (CI) of recurrence-free survival prediction models based on the Oncotype DX, the MammaPrint, and the E2F4 score, respectively. (**B**) The prediction accuracy (AUC) of the corresponding PPMs. (**C**) Application of models trained from the Curtis discovery data. (**D**) Application of models trained from the Curtis validation data. For each patient a prognostic score (*P*-score) and a confidence score (C-score) were predicted by the CPM and the PPM, respectively. Patients were sorted in the decreasing order of their C-scores, and the average concordance of the CPM in the top N patients were calculated from *N* = 1 to all patients. The curve was smoothed by averaging values within a sliding window of size 200.

Next, we extended the above-analysis to the MammaPrint and the E2F4 signatures. Specifically, we used the CPM/PPM trained from the Curtis discovery or validation dataset to another breast cancer dataset compiled by Ur Rehman et al.^26^. The original Ur Rehman dataset contains 1570 breast cancer gene expression profiles, from which we selected 880 profiles with recurrence-free survival information. In the PPM, we used a subset of clinical variables that were shared by the Curtis and the Ur Rehman data, including age, tumor size, ER status, grade, lymph node status, and PAM50 subtype. For each patient in the Ur Rehman data, we calculated a *P*-score, a C-score, and sample-specific concordance. We performed the same analyses for the Oncotype DX, the MammaPrint and the E2F4 signatures. Our results are summarized in Fig. 6. As shown, the Cox regression-based CPM trained from the Curtis discovery data achieves a concordance index of 0.679 for Oncotype DX, 0.680 for MammaPrint, and 0.638 for the E2F4 signature, respectively (Fig. 6A). The PPM model trained from the Curtis discovery data achieves an AUC score of 0.651, 0.624, and 0.646, respectively (Fig. 6B). As shown in Fig. 6C and D, patients with higher C-scores are associated with higher average concordance for all three multi-gene signatures. These results confirmed the effectiveness of the CPM and the PPM trained from Curtis data in the independent Rehman dataset, supporting the potential clinical application of them.

## Discussion

In this study, we propose a new framework to improve the clinical application of existing cancer prognostic and predictive biomarkers. According to this framework, to apply a biomarker, two models (CPM and PPM) will be used to predict the clinical outcome of a patient and to evaluate the confidence of the prediction, simultaneously. High confidence scores (e.g., C-score > 0.8) from the PPM indicate high applicability of the CPM to patients, and for them clinical decision can be more confidently made based on the predicted clinical outcomes. In contrast, patients with low confidence scores (e.g., C-score < 0.2) should be more carefully interpreted. Patient applicability of a CPM is determined by a combination of various factors including clinical features and genomic features of tumor samples. By using this framework we expect to improve the precise application of existing biomarkers for aiding physicians in decision-making.

This framework may pose positive impact on the development of new cancer biomarkers. A large number of candidate biomarkers have been proposed in previous studies, however, over 99% of them failed to introduce clinical applications^20^. Heterogeneity between tumors from different patients represents one of the important reasons that lead to such a failure rate. A biomarker developed in a specific discovery cohort may not applicable to another cancer dataset generated from a different patient population, resulting in a low reproducibility. The PPM model can be used to determine the clinical and genomic features that define a cancer subset for which the investigated biomarker is effective. Therefore, jointly developing the CPM and PPM for a biomarker will increase its success rate for clinical application.

Limited by the availability of clinical features, we used many overlapping predictors in the CPM and the PPM in the neoadjuvant response prediction examples. In principle, we would expect a different set of clinical/genomic features that determine the accuracy of the CPM and PPM models. In the era of precision medicine, it will become common in the future to profile the somatic mutations of each cancer patient in a panel of cancer genes or using whole exome/genome sequencing. The generation of other types of “omics” data for cancer patients, such as epigenomic and proteomic data, might also become routine analyses. To identify and include genomic features that are associated with the CPM applicability to patients will further improve precision of PPM in identifying predictable patients.

In this study, we formularized the PPMs as a classification problem using the random forest method. In fact, other statistical or machine-learning techniques (e.g. logistic regression, support vector machine) can be readily used in this framework. In fact, we have shown that using random survival forests for survival prediction (instead of Cox proportional hazards) also performs well, and the PPM is still able to delineate which samples have higher concordance of prediction. Moreover, we can also define PPMs as a regression model. For instance, instead of classifying well- versus poorly-predicted patients using a classification model, we can define a regression-based model to predict the sample-specific concordance, and use this PPM to calculate the confidence score of patients. Finally, it would be fairly straightforward to extend this framework to improve clinical application of biomarkers to non-cancer human diseases.

In this study, we implement two distinct models, clinical outcome precision model (CPM) and predictability prediction model (PPM), to predict patients’ prognosis or diagnosis (pCR vs RD) by classification or cox regression and then predict the biomarker’s applicability to patients. We validate our framework with three widely used clinical biomarkers (Oncotype DX, MammaPrint and E2F4) for breast cancer, which we successfully discover those biomarkers have higher accuracy of predictability within a subset of patient with specific clinical or genetic features. The c-score generated from the predictability prediction model (PPM) help us evaluate how well a biomarker can be applicable to a patient which has great potential to be utilized in drug or biomarker clinical trials. By applying the framework, researchers can systematically select patients with high c-score to enter the clinical trial which may revive clinically failed drugs and biomarkers.

## Materials and methods

### Breast cancer microarray datasets

The following breast cancer gene expression datasets were used in this study: the Curtis dataset (METABRIC)^25^, the Ur-Rehman dataset (GSE47561)^26^, the Hatzis dataset (GSE25066)^24^, the GSE20194 dataset^27^, and the GSE22093 dataset^28^. All except for the Curtis dataset were downloaded from the GEO (Gene Expression Omnibus) database^29^ using the corresponding GEO accession IDs. The Curtis dataset was downloaded from the European Genome Phenome Archive with accession ID EGAS00000000083. This dataset contains gene expression for 1992 breast cancer patients (997 in the discovery and 995 in the validation cohorts) measured using the Illumina HT-12 v3 platform (Illumina_Human_WG-v3). The Ur-Rehman dataset is a meta-dataset that combines 10 breast cancer datasets, containing a total of 1570 tumor samples measured by Affymetrix microarray platform. Among these samples, 880 samples had relapse-free survival and other required clinical information, and were used in our analysis^26^. The Hatzis dataset contains gene expression profiles for 508 invasive breast tumor samples that were collected by fine needle aspiration (FNA) or core biopsy (CBX) prior to any systemic therapy. All of these patients were then treated by neoadjuvant taxane-anthracycline chemotherapy and followed to determine the treatment efficacy– pCR or RD^24^. Gene expression was profiled using the Affymetrix Human Genome U133A Array platform (GPL96). The GSE20194 dataset and the GSE22093 dataset contain gene expression profiles for 230 and 103 breast cancers, respectively, that were generated from fine needle aspiration specimens of newly diagnosed patients before any therapy. Patients were treated with neoadjuvant chemotherapy using paclitaxel, 5-fluorouracil, cyclophosphamide and doxorubicin. After treatment, patients were then subject to surgical resection and their response to neoadjuvant chemotherapy was assessed. Both datasets were generated from the Affymetrix Human Genome U133A Array platform (GPL96).

All these datasets were generated from one-channel microarray platforms. Datasets from the GEO database were downloaded as processed data, providing absolute expression at the probeset level. We converted these data into gene expression data by mapping probeset IDs to gene symbols according to the Affymetrix annotation files. If a gene had multiple probesets, the probeset with the highest average intensity across all samples was used to represent this gene. All datasets used in this study are freely accessible and do not require any institutional and/or licensing committee approving the experiments. All experiments were performed in accordance with relevant guidelines and regulations.

### Calculation of recurrence scores using three multi-gene signatures

Given a breast cancer dataset, the Oncotype DX and MammaPrint scores were calculated for all samples by using the genefu R package^30^. The R functions “oncotypedx” and “gene70” were used for Oncotype DX and MammaPrint signatures, respectively. The E2F4 signature consists of a total of 199 target genes that are regulated by the cell cycle regulatory transcription factor E2F4^23^. A 33-gene subset of the original 199 target genes have been optimized for clinical use^21^. The 33-gene signature is deposited in Suppl. Table 1. This signature was used to calculate an E2F4 score that indicated the regulatory activity of E2F4 in a breast cancer sample. The E2F4 score has been shown to be predictive of patient prognosis and response to neoadjuvant chemotherapy in breast cancer^21,23^.

### Determination of PAM50 molecular subtypes

Breast cancer has five major molecular subtypes including Basal-like, Her2-enriched, LumA, LumB, and Normal-like^31,32^. The molecular subtypes of samples in the Curtis and Hatzis breast cancer data have been provided by the original publications. For other datasets, we determined the molecular subtype by using the genefu R package^30^. In this package, a PAM50 centroid profile was provided for each of the five breast cancer subtypes, consisting of an expression signature of 50 discriminative genes. Given a breast cancer dataset, the expression levels (log2 intensity) of these 50 genes were selected, median normalized across all samples, and correlated with the PAM50 centroid profiles to obtain the Spearman correlation coefficients of each sample with the five subtypes. A sample is classified as the molecular subtype with the maximal correlation.

### Prediction of patient response to neoadjuvant therapy in breast cancer

Oncotype DX, MammaPrint and the E2F4 signature have been previously shown to be predictive of patient response to neoadjuvant chemotherapy in breast cancer^16,21^. In this study, we applied the random forest statistical learning method^33^ to construct classification models to predict patient response and to predict pCR versus RD. We used 10,000 trees to train the PPM model. In general, random forest models tend to perform more robustly with more trees at the cost of computational time. We tested the prediction accuracy of the random forest model with 100, 1000, 10,000, and 100,000 trees, and chose to use 10,000 trees since there was only a marginal increase in accuracy with 100,000 trees compared to 10,000 but a drastically longer training time. Other hyperparameters were left as the default values in the random forest R package. The predictors used in these models include age, ER-status, tumor stage, and the score calculated based on one of the three signatures. We call these models clinical outcome prediction models (Classification-CPMs) to discriminate them from the models used for determining the predictability of samples—predictability prediction models (PPMs). In a neoadjuvant therapy dataset, the number of pCR patients is typically much less than the number of RD patients, introducing the so-called imbalanced class problem. When an imbalanced dataset is used, a classification model may simply predict all samples to be the dominant class. To address this problem, we down-sampled the RD patients to obtain a balanced dataset by randomly removing RD patients until the number of RD patients matched the number of pCR patients. Our classification models achieved comparable prediction accuracy rate for pCR and RD patients, indicating the effectiveness of this down-sampling strategy.

Ten-fold cross-validation was used to estimate the performance of our classification models. Specifically, pCR and RD samples in a dataset were randomly divided into 10 groups of each size. Each time, the model was trained using a pooled dataset containing 9 groups of pCR and RD samples, and applied to the remaining samples. Then the training groups and the test group were rotated, until all samples had been predicted once. The predicted classes were then compared with the actual response of patients to determine the ROC (Receiver Operating Characteristic) curve and calculate the AUC (Area Under Curve) score. This procedure was performed 10 times and the average AUC score was used to represent the cross-validation accuracy of the model.

The relative importance of a predictor in a random forest classification model indicates its contribution to class prediction. To calculate the relative importance, a predictor of interest is excluded from the full model to examine the decrease of classification accuracy. The R package “RandomForest” was used to implement these models and analyses.

### Calculation of sample-specific confidence scores of classification models

Due to the heterogeneity of cancers, a CPM may not be effective for all tumor samples. Given a particular CPM, we constructed a random forest-based classification model to predict its applicability to different patients based on their clinical and genomic features. Specifically, in the context of neoadjuvant therapy response prediction, we used the Hatzis data to construct the predictability prediction model (PPM). First, we divided all patients into correctly- and incorrectly-predicted groups by comparing the outcomes predicted by an above-described CPM (e.g., the Oncotype DX model). The sample sizes in these groups are balanced using random down-sampling on the group with more samples (the correctly-predicted group) to match the group with less samples. The Oncotype DX PPM was trained with *N* = 64 samples in each group, MammaPrint with *N* = 61 samples in each group, and E2F4 with *N* = 61 samples in each group. Then, we constructed a random forest model to classify the two patient groups and the prediction accuracy of this PPM was assessed by tenfold cross-validation method. Finally, this PPM was used on other datasets (GSE20194 and GSE22093) to further evaluate its performance. The PPM models output the probability of a patient to be a “correct” prediction. This probability indicates the applicability of the corresponding CPM to a patient, providing a confidence score of the patient. Note that we used random forest as the backbone model for the PPM as an example of its feasibility, but this framework could extended to other methods and models.

### Prediction of recurrence-free survival of patients with breast cancer

Univariate Cox proportional hazards models (Cox regression-CPMs) were used to apply Oncotype DX, MammaPrint and the E2F4 scores to predict patient recurrence-free survival in breast cancer. The default parameters for the coxph function in the R package “survival” were used: zero is the initial value for all variables, the Efron approximation is used to resolve ties, and columns that are linear combinations of previous columns are skipped. P-values for the Cox models were estimated using the Wald test. To model potential nonlinear associations between the predictors and the clinical outcome, we also used random survival forests to predict survival with the rfsrc function from the R package randomForestSRC^34^. We used the default parameters for fitting the random forest survival predictor, which includes 500 trees.

### Calculation of sample-specific confidence scores of Cox regression models

Given a Cox regression or random forest CPM implementing a survival prediction biomarker, the following procedure was used to construct PPMs to calculate confidence scores of each patient: First, the CPM was applied to the Curtis data and for each patient a sample-specific concordance was calculated. For each patient, the survival time can be compared with all the other patients in the dataset. However, given a pair of patients with survival information (t_1_, e_1_) and (t_2_, e_2_), only the following scenarios will results in an effective comparison: e_1_ = e_2_ = 1 (both patients have recurrence event); t_1_ > t_2_ and e_1_ = 0 and e_2_ = 1 (patient 2 but not patient 1 has a recurrence event, and recurrence-free survival of patient 2 is shorter than the follow-up time of patient 1); and vice versa, t_1_ < t_2_ and e_1_ = 1 and e_2_ = 0. The sample-specific concordance for a patient is the proportion of effective pairwise comparisons that are correctly predicted by the Cox regression model. By using the overall concordance as the threshold, patients were then divided into a well-predicted group and a poorly-predicted group. Second, we constructed PPM using the random forest method to classify the two patient groups. Finally, this PPM was used to calculate the confidence scores of patients in a dataset.

## Supplementary Information

Supplementary Information 1.

Supplementary Information 2.

## Data Availability

The Ur-Rehman and Hatzis dataset can be downloaded from the GEO repository, under accession IDs GSE47561 and GSE25066, respectively. Breast cancer patients’ responses to neoadjuvant therapy can also be downloaded from GEO under accession IDs GSE20194 and GSE22093. The Curtis breast cancer dataset can be downloaded from the European Genome Phenome Archive with accession number EGAS00000000083. All code used for analysis in this report are deposited in https://github.com/ksyao2002/Applicability-gene-signatures.
