# Supplementary material for: A framework to predict the applicability of Oncotype DX, MammaPrint, and E2F4 gene signatures for improving breast cancer prognostic prediction
Source: Sci Rep. 2022 Feb 9;12:2211. doi: 10.1038/s41598-022-06230-7 (PMC8828770; doi:10.1038/s41598-022-06230-7)
Supplement: Supplementary file 1 — Supplementary Information 1. [file 41598_2022_6230_MOESM1_ESM.docx]

A framework to predict the applicability of Oncotype DX, MammaPrint, and E2F4 gene signatures for improving breast cancer prognostic prediction

Kevin Yao^1^, Chun-Yip Tong^2^, Chao Cheng^2,3,4*^

1. Department of Electrical and Computer Engineering, Texas A&M University, College Station, TX

2. Department of Medicine, Baylor College of Medicine, Houston, TX 77030, USA
3. Dan L Duncan Comprehensive Cancer Center, Baylor College of Medicine, Houston, TX 77030, USA

4. Institute for Clinical and Transcriptional Research, Baylor College of Medicine, Houston, TX 77030, USA

*Corresponding author

Email: chao.cheng@bcm.edu

Supplementary materials


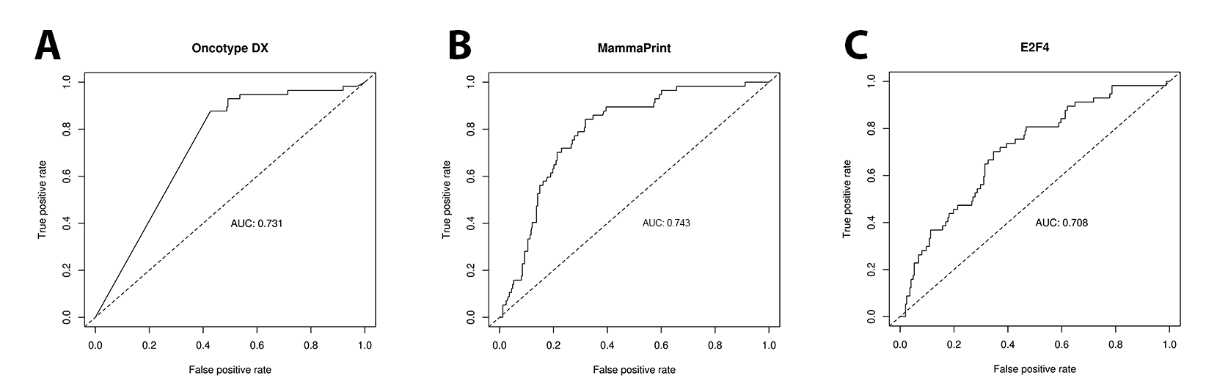


Supplementary Figure S1. (A-C) ROC curves showing the AUC of Oncotype DX, MammaPrint, and E2F4 scores respectively used as univariate predictors of response to neoadjuvant chemotherapy. Generally, incorporating clinical variables in the random forest model increases the performance metric of the model.


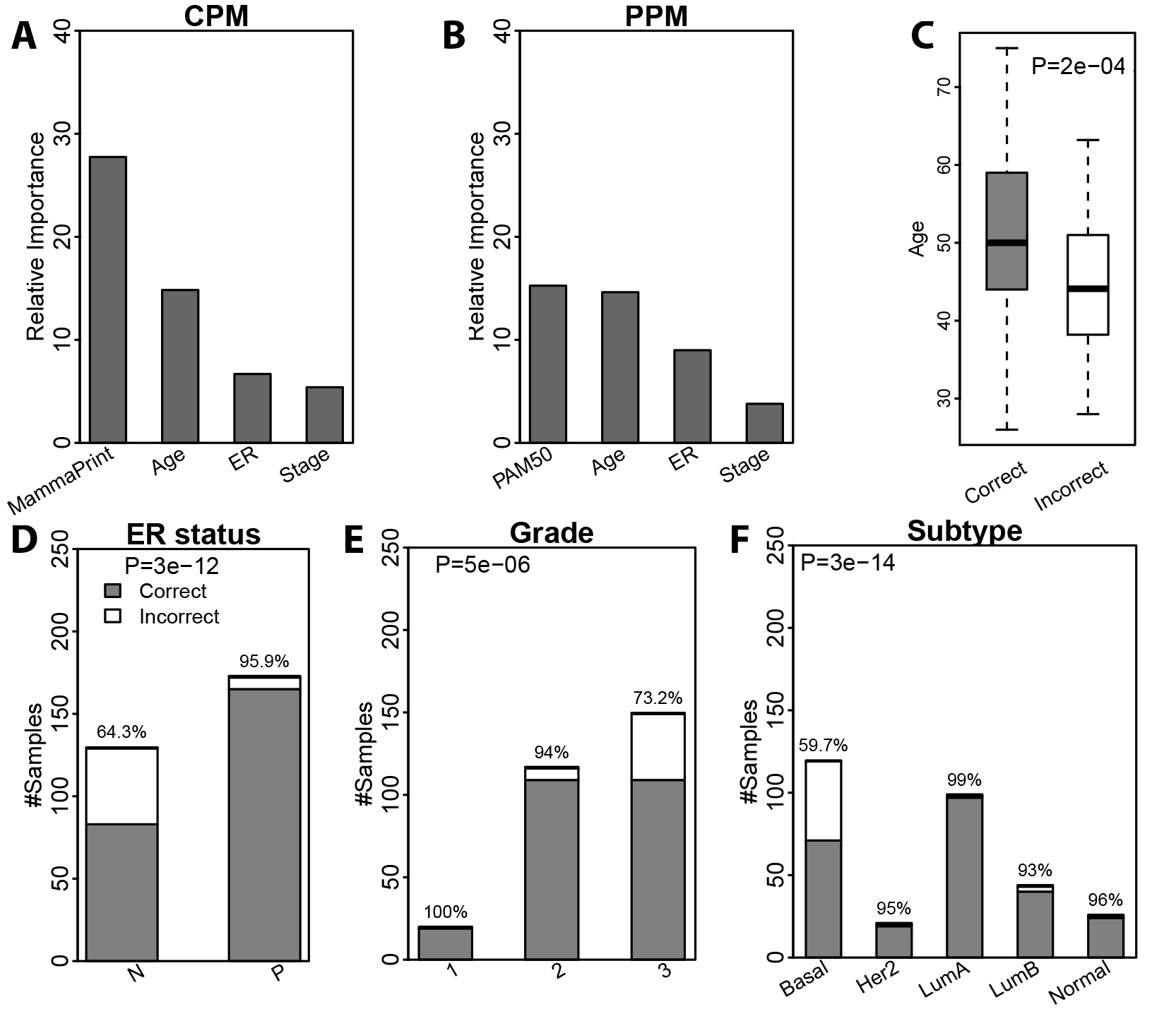


Supplementary Figure S2. Association of predictions based on the MammaPrint assay with clinical variables. (A, B) Relative importance of predictors in the CPM and PPM, respectively. (C) Older patients are more likely to be predicted correctly. (D) ER positive patients are more likely to be predicted correctly. (E) Patients with lower grade are more likely to be predicted correctly. (F) Association of molecular subtypes with prediction accuracy.


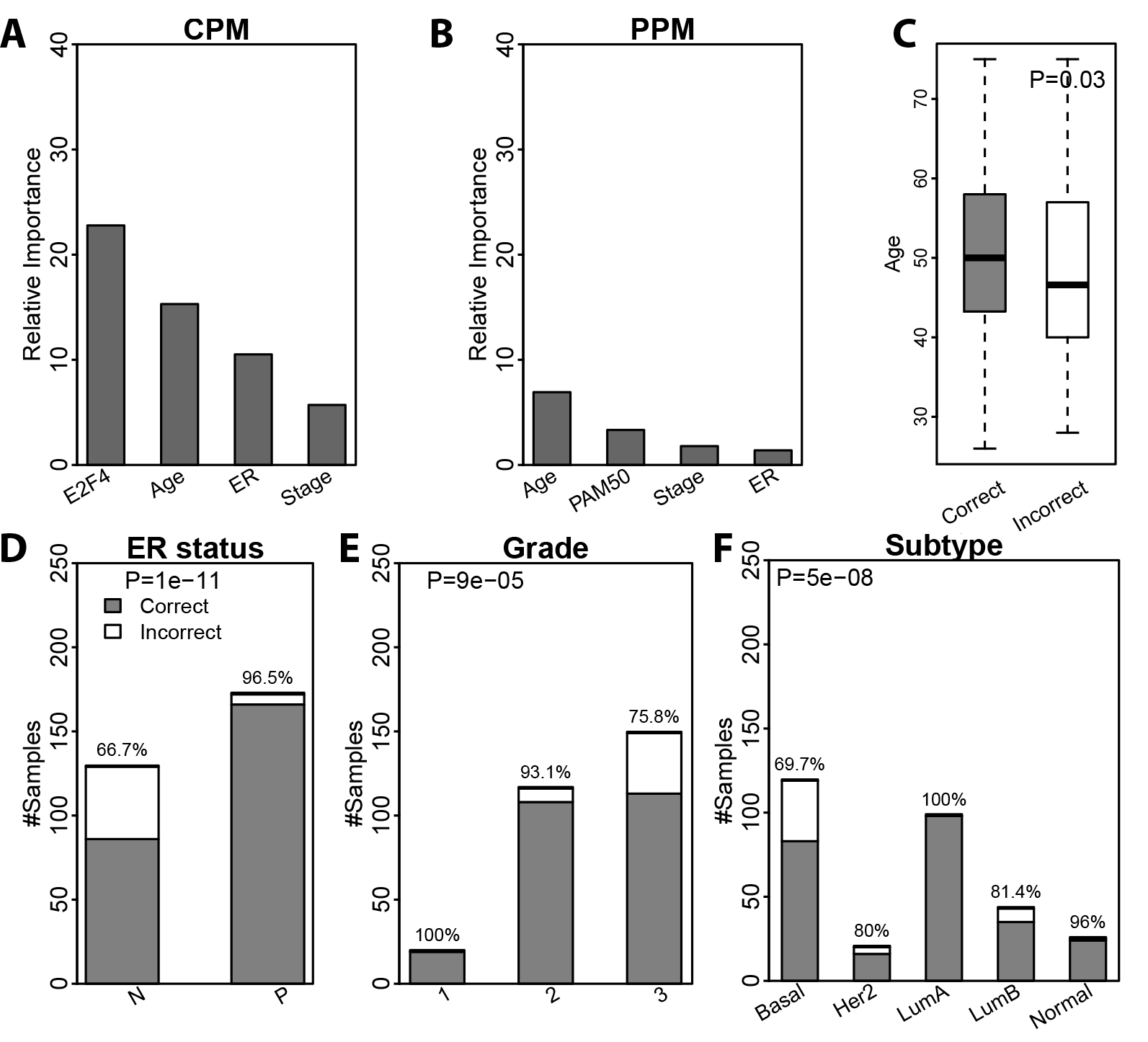


Supplementary Figure S3. Association of predictions based on the E2F4 assay with clinical variables. (A, B) Relative importance of predictors in the CPM and PPM, respectively. (C) Older patients are more likely to be predicted correctly. (D) ER positive patients are more likely to be predicted correctly. (E) Patients with lower grade are more likely to be predicted correctly. (F) Association of molecular subtypes with prediction accuracy.


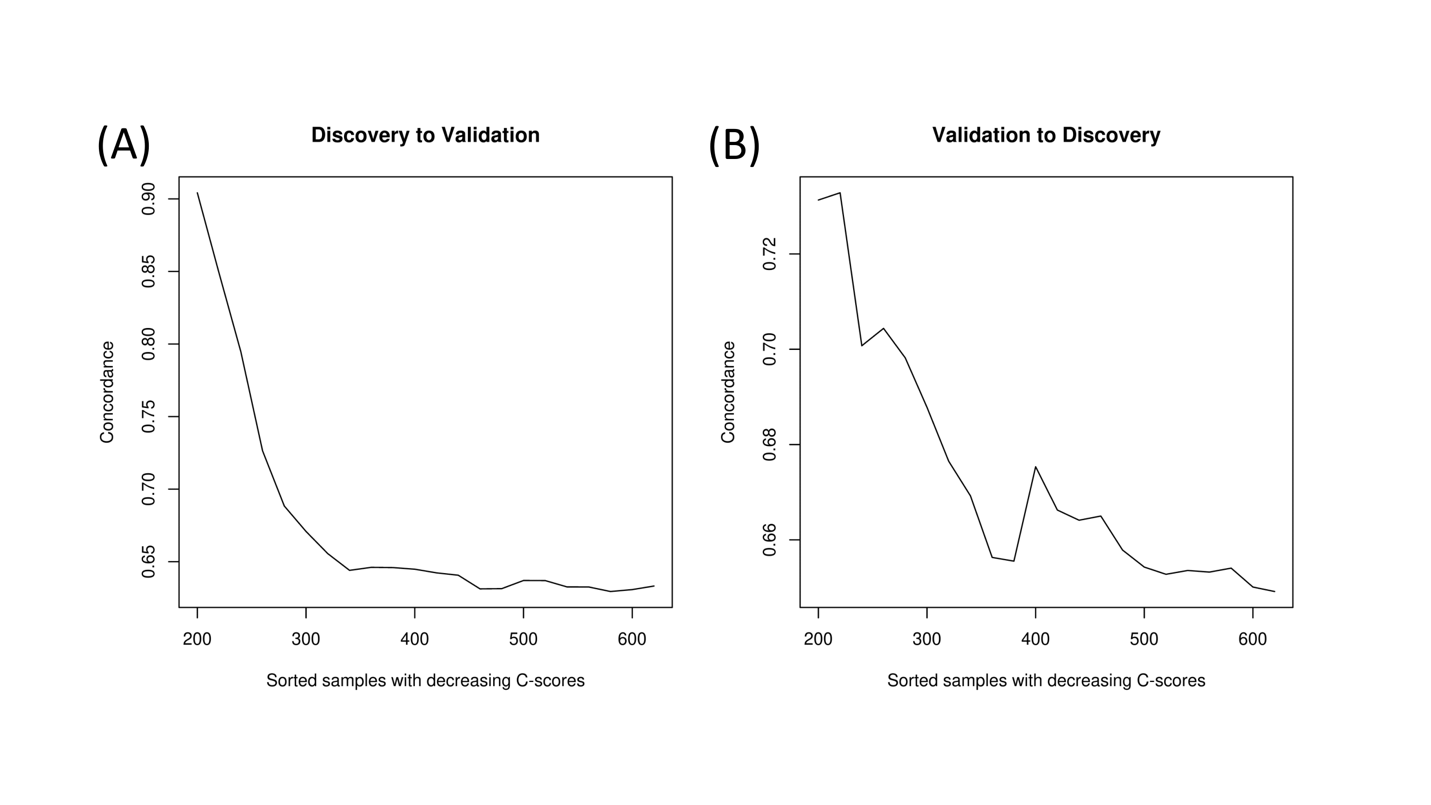


Supplementary Figure S4. Concordance of a CPM that utilizes random survival forest instead of Cox proportional hazards as the predictor of survival. (A) Application of the CPM/PPMs trained from the discovery data to patients in the validation cohort, and (B) vice versa. Patients are sorted in the decreasing order of their confidence scores, and the average concordance in the top N patients was calculated from N=1 to all patients. The curve was smoothed by averaging values within a sliding window of size 200.

| *Gene* | *Weight* |
| --- | --- |
| BIRC5 | 0.182904 |
| NUF2 | 0.21915 |
| SGOL1 | 0.137376 |
| CDCA3 | 0.136733 |
| KIF20A | 0.175755 |
| NUSAP1 | 0.160178 |
| OIP5 | 0.170259 |
| KIF23 | 0.171274 |
| PRC1 | 0.159252 |
| BUB1 | 0.226243 |
| CDCA8 | 0.165218 |
| UBE2T | 0.17157 |
| CEP55 | 0.234161 |
| NCAPH | 0.140164 |
| KIF15 | 0.179671 |
| FOXM1 | 0.170847 |
| EXO1 | 0.16773 |
| CENPA | 0.196286 |
| AURKB | 0.11828 |
| GTSE1 | 0.12707 |
| CDCA5 | 0.178454 |
| C15orf42 | 0.169853 |
| BLM | 0.145058 |
| ASPM | 0.173875 |
| RAD51 | 0.168714 |
| NDC80 | 0.17913 |
| DEPDC1B | 0.196929 |
| NEIL3 | 0.185208 |
| STIL | 0.141571 |
| RAD54L | 0.129884 |
| CENPF | 0.198774 |
| PLK1 | 0.099492 |
| KIF2C | 0.192363 |
| BUB1B | 0.176422 |

Supp. Table S1. Gene list with weights for the E2F4 gene signature.

| **Gene signature** | **Genes** |
| --- | --- |
| *Oncotype DX* | *Ki67, STK15, BIRC5, CCNB1, MYBL2, MMP11, CTSL2, GRB7, HER2, ER, PGR, BCL2, SCUBE2, GSTM1, BAG1, CD68, ACTB, GAPDH, GUS, RPLPO, TFRC* |
| *MammaPrint* | *BBC3, EGLN1, TGFB3, ESM1, IGFBP5, FGF18, SCUBE2, TGFB3, WISP1, FLT1, HRASLS, STK32B, RASSF7, DCK, MELK, EXT1, GNAZ, EBF4, MTDH, PITRM1, QSCN6L1, CCNE2, ECT2, CENPA, LIN9, KNTC2, MCM6, NUSAP1, ORC6L, TSPYL5, RUNDC1, PRC1, RFC4, RECQL5, CDCA7, DTL, COL4A2, GPR180, MMP9, GPR126, RTN4RL1, DIAPH3, CDC42BPA, PALM2, ALDH4A1, AYTL2, OXCT1, PECI, GMPS, GSTM3, SLC2A3, FLT1, FGF18, COL4A2, GPR180, EGLN1, MMP9, LOC100288906, C9orf30, ZNF533, C16orf61, SERF1A, C20orf46, LOC730018, LOC100131053, AA555029_RC, LGP2, NMU, UCHL5, JHDM1D, AP2B1, MS4 A7, RAB6B* |
| *E2F4* | *BIRC5, NUF2, SGOL1, CDCA3, KIF20A, NUSAP1, OIP5, KIF23, PRC1, BUB1, CDCA8, UBE2T, CEP55, NCAPH, KIF15, FOXM1, EXO1, CENPA, AURKB, GTSE1, CDCA5, C15orf42, BLM, ASPM, RAD51, NDC80, DEPDC1B, NEIL3, STIL, RAD54L, CENPF, PLK1, KIF2C, BUB1B* |
| *Prosigna PAM50* | *UBE2T, BIRC5, NUF2, CDC6, CCNB1, TYMS, MYBL2, CEP55, MELK, NDC80, RRM2, UBE2C, CENPF, PTTG1, EXO1, ORC6L, ANLN, CCNE1, CDC20, MKI67, KIF2C, ACTR3B, MYC, EGFR, KRT5, PHGDH, CDH3, MIA, KRT17, FOXC1, SFRP1, KRT14, ESR1, SLC39A6, BAG1, MAPT, PGR, CXXC5, MLPH, BCL2, MDM2, NAT1, FOXA1, BLVRA, MMP11, GPR160, FGFR4, GRB7, TMEM45B, ERBB2* |

Supplementary Table S2. Gene lists of the Oncotype DX, MammaPrint, E2F4, and Prosigna PAM50 gene signatures. There are a few genes shared between Oncotype DX/MammaPrint/E2F4: one shared gene between Oncotype DX and MammaPrint (SCUBE2), one shared between Oncotype DX and E2F4 (BIRC5), and three shared between MammaPrint and E2F4 (CENPA, NUSAP1, and PRC1).
