# Supplementary material for: A framework to predict the applicability of Oncotype DX, MammaPrint, and E2F4 gene signatures for improving breast cancer prognostic prediction
Source: Sci Rep. 2022 Feb 9;12:2211. doi: 10.1038/s41598-022-06230-7 (PMC8828770; doi:10.1038/s41598-022-06230-7)
Supplement: Supplementary file 2 — Supplementary Information 2. [file 41598_2022_6230_MOESM2_ESM.docx]

A framework to predict the applicability of gene signatures for improving prognostic prediction

Kevin Yao^1^, Chun-Yip Tong^2^, Chao Cheng^2,3,4*^

1. Department of Electrical and Computer Engineering, Texas A&M University, College Station, TX

2. Department of Medicine, Baylor College of Medicine, Houston, TX 77030, USA
3. Dan L Duncan Comprehensive Cancer Center, Baylor College of Medicine, Houston, TX 77030, USA

4. Institute for Clinical and Transcriptional Research, Baylor College of Medicine, Houston, TX 77030, USA

*Corresponding author

Email: chao.cheng@bcm.edu

Supplementary Tables.

| *Gene* | *Weight* |
| --- | --- |
| BIRC5 | 0.182904 |
| NUF2 | 0.21915 |
| SGOL1 | 0.137376 |
| CDCA3 | 0.136733 |
| KIF20A | 0.175755 |
| NUSAP1 | 0.160178 |
| OIP5 | 0.170259 |
| KIF23 | 0.171274 |
| PRC1 | 0.159252 |
| BUB1 | 0.226243 |
| CDCA8 | 0.165218 |
| UBE2T | 0.17157 |
| CEP55 | 0.234161 |
| NCAPH | 0.140164 |
| KIF15 | 0.179671 |
| FOXM1 | 0.170847 |
| EXO1 | 0.16773 |
| CENPA | 0.196286 |
| AURKB | 0.11828 |
| GTSE1 | 0.12707 |
| CDCA5 | 0.178454 |
| C15orf42 | 0.169853 |
| BLM | 0.145058 |
| ASPM | 0.173875 |
| RAD51 | 0.168714 |
| NDC80 | 0.17913 |
| DEPDC1B | 0.196929 |
| NEIL3 | 0.185208 |
| STIL | 0.141571 |
| RAD54L | 0.129884 |
| CENPF | 0.198774 |
| PLK1 | 0.099492 |
| KIF2C | 0.192363 |
| BUB1B | 0.176422 |

Supp. Table S1. Gene list with weights for the E2F4 gene signature.

| **Gene signature** | **Genes** |
| --- | --- |
| *Oncotype DX* | *Ki67, STK15, BIRC5, CCNB1, MYBL2, MMP11, CTSL2, GRB7, HER2, ER, PGR, BCL2, SCUBE2, GSTM1, BAG1, CD68, ACTB, GAPDH, GUS, RPLPO, TFRC* |
| *MammaPrint* | *BBC3, EGLN1, TGFB3, ESM1, IGFBP5, FGF18, SCUBE2, TGFB3, WISP1, FLT1, HRASLS, STK32B, RASSF7, DCK, MELK, EXT1, GNAZ, EBF4, MTDH, PITRM1, QSCN6L1, CCNE2, ECT2, CENPA, LIN9, KNTC2, MCM6, NUSAP1, ORC6L, TSPYL5, RUNDC1, PRC1, RFC4, RECQL5, CDCA7, DTL, COL4A2, GPR180, MMP9, GPR126, RTN4RL1, DIAPH3, CDC42BPA, PALM2, ALDH4A1, AYTL2, OXCT1, PECI, GMPS, GSTM3, SLC2A3, FLT1, FGF18, COL4A2, GPR180, EGLN1, MMP9, LOC100288906, C9orf30, ZNF533, C16orf61, SERF1A, C20orf46, LOC730018, LOC100131053, AA555029_RC, LGP2, NMU, UCHL5, JHDM1D, AP2B1, MS4 A7, RAB6B* |
| *E2F4* | *BIRC5, NUF2, SGOL1, CDCA3, KIF20A, NUSAP1, OIP5, KIF23, PRC1, BUB1, CDCA8, UBE2T, CEP55, NCAPH, KIF15, FOXM1, EXO1, CENPA, AURKB, GTSE1, CDCA5, C15orf42, BLM, ASPM, RAD51, NDC80, DEPDC1B, NEIL3, STIL, RAD54L, CENPF, PLK1, KIF2C, BUB1B* |
| *Prosigna PAM50* | *UBE2T, BIRC5, NUF2, CDC6, CCNB1, TYMS, MYBL2, CEP55, MELK, NDC80, RRM2, UBE2C, CENPF, PTTG1, EXO1, ORC6L, ANLN, CCNE1, CDC20, MKI67, KIF2C, ACTR3B, MYC, EGFR, KRT5, PHGDH, CDH3, MIA, KRT17, FOXC1, SFRP1, KRT14, ESR1, SLC39A6, BAG1, MAPT, PGR, CXXC5, MLPH, BCL2, MDM2, NAT1, FOXA1, BLVRA, MMP11, GPR160, FGFR4, GRB7, TMEM45B, ERBB2* |

Supplementary Table S2. Gene lists of the Oncotype DX, MammaPrint, E2F4, and Prosigna PAM50 gene signatures. There are a few genes shared between Oncotype DX/MammaPrint/E2F4: one shared gene between Oncotype DX and MammaPrint (SCUBE2), one shared between Oncotype DX and E2F4 (BIRC5), and three shared between MammaPrint and E2F4 (CENPA, NUSAP1, and PRC1).
